# Management and outcomes of congenital pulmonary airway malformations and bronchopulmonary sequestrations diagnosed in the fetal period

**DOI:** 10.1007/s00383-026-06610-4

**Published:** 2026-09-01

**Authors:** Andrea L. Corso, Isadora L. Flores, Marina C. Tonietto, Victorio S. Boff, Luciano F. Schopf, Paola B. Isolan, Clarice B. Giacomini, Jose A. Magalhães, Jose Carlos Fraga

**Affiliations:** 1 Neonatology Service, Hospital de Clinicas de Porto Alegre (HCPA), Porto Alegre, Brazil; 2 Department of Pediatrics, Faculty of Medicine, Federal University of Rio Grande do Sul (UFRGS), Porto Alegre, RS Brazil; 3 School of Medicine, Luterana University of Brazil (ULBRA), Canoas, Brazil; 4 School of Medicine, Caxias do Sul University of Brazil (UCS), Caxias do Sul, Brazil; 5 Pediatric Surgery Service, Hospital de Clinicas de Porto Alegre (HCPA), Porto Alegre, Brazil; 6 Department of Surgery, Faculty of Medicine, Federal University of Rio Grande do Sul (UFRGS), Porto Alegre, RS Brazil; 7 Fetal Medicine Group, Gynecology and Obstetric Service, Hospital de Clinicas de Porto Alegre (HCPA), Porto Alegre, Brazil; 8 Department of Gynecology and Obstetrics, Faculty of Medicine, Federal University of Rio Grande do Sul (UFRGS), Porto Alegre, RS Brazil; 9 Pediatric Thoracic and Airway Surgery Section, Hospital de Clinicas de Porto Alegre (HCPA), Porto Alegre, Brazil; 10 Pediatric Surgery, Federal University of Rio Grande do Sul (UFRGS) Hospital de Clinicas de Porto Alegre, Rua Ramiro Barcelos, 2350, sala 600, Porto Alegre, RS 90035-903 Brazil

**Keywords:** Pulmonary congenital malformations, Congenital pulmonary airway malformation, Bronchopulmonary sequestration, Fetus, Intrauterine diagnoses

## Abstract

**Purpose:**

To describe the prenatal and postnatal management and outcomes of fetuses prenatally diagnosed with congenital pulmonary airway malformation (CPAM) or bronchopulmonary sequestration (BPS).

**Methods:**

This retrospective study included patients with a prenatal ultrasonographic diagnosis of CPAM or BPS who were treated at a quaternary referral hospital between January 2014 and December 2024.

**Results:**

Twenty-seven fetuses were diagnosed with CPAM or BPS by prenatal ultrasonography. Five (18.5%) showed complete regression of the lesions during gestation. Among the 22 fetuses with persistent malformations at birth, 12 (54.5%) had left-lung malformations and 10 (45.5%) had right-lung malformations. At birth, 12 neonates (54.5%) were asymptomatic, and 10 (45.5%) had respiratory symptoms. Twenty patients underwent surgical resection of the lesion: 15 by thoracoscopy and five by open surgery. There were no deaths, and postoperative outcomes were excellent over a follow-up period of 18 (10–33.5) months.

**Conclusion:**

Prenatal diagnosis of CPAM or BPS allows appropriate management of these malformations during gestation and after birth. Ultrasonographic follow-up throughout pregnancy is essential because lesions may either regress or increase in size and cause severe fetal complications. Surgical resection was performed in both symptomatic and asymptomatic children, with no deaths and excellent outcomes.

## Introduction

The advent of routine ultrasound screening during pregnancy has led to an increase in the detection of congenital lung malformations (CLMs), a heterogeneous group of lesions involving the lung parenchyma and its bronchovascular structures that account for 5–18% of all congenital anomalies [1]. The currently reported incidence of CLMs—1 in 2,500 births—is probably still underestimated, given that some lesions are not detected or are not diagnosed until later in life [2]. Although the etiology of CLMs is not yet fully understood, these anomalies are believed to have a common embryological origin, with the location of the lesion and the gestational age at which it develops influencing the resulting malformation [3].

Among CLMs, congenital pulmonary airway malformation (CPAM) and bronchopulmonary sequestration (BPS) are the most common [3]. CPAM is characterized by a hamartomatous proliferation of tissue that forms cystic spaces lined by respiratory epithelium, which communicate with the bronchial tree and receive their blood supply from the pulmonary artery [4]. It may present as multiple cysts, a single cyst, or a solid mass with or without small cysts [3]. The incidence of CPAM has historically been estimated to range from 1 in 11,000 to 1 in 35,000 births, but a recent prospective study suggested an incidence of 1 in 7,200 births [3]. BPS consists of a nonfunctioning lung lesion that does not communicate with the tracheobronchial tree and receives an anomalous arterial blood supply from the systemic circulation, most typically from the thoracic or abdominal aorta [4–7]. BPS may appear as a solid or microcystic lesion and may occur either within a lung lobe (intralobar) or outside the lung parenchyma (extralobar). BPS has an estimated incidence of 1 in 20,000 births [3]. The co-occurrence of CPAM and BPS is known as a hybrid lesion [8].

Major associated malformations occur in 5% to 10% of newborns with CLMs. CPAM and BPS are most frequently associated with heart disease and gastrointestinal malformations [9]. Prenatal diagnosis of CPAM and BPS is thus essential for appropriate management of these patients, as it provides an opportunity to refer pregnant women to specialized centers for follow-up and assessment of the need for drug treatment or even invasive intrauterine procedures [10]. In the present study, we present a retrospective analysis of the prenatal and postnatal management and outcomes of fetuses prenatally diagnosed with CPAM or BPS at a quaternary hospital.

## Methods

For this retrospective study, we reviewed medical records to retrieve information on the prenatal diagnosis of CPAM and BPS and the follow-up of affected fetuses at Hospital de Clínicas de Porto Alegre (HCPA), a quaternary hospital in southern Brazil. The study covered the period from January 2014 to December 2024 and was approved by the Institutional Research Ethics Committee under approval number 2023 − 0466 (CAAE No. 76684623.4.0000.5327). Information was obtained from the following services: obstetrics and gynecology (fetal medicine section), neonatology, and pediatric surgery (thoracic surgery section). The variables evaluated were sex, gestational age (GA) at diagnosis and birth, the prenatal and postnatal types and locations of the lesions, clinical manifestations, postnatal complications, associated malformations, surgical procedures, age and weight at surgery, surgical complications, and status at follow-up. When sufficient data were available, prenatal CPAM growth was monitored by calculating the CPAM volume ratio (CVR).

The pregnant women were referred to our service—a center of excellence in fetal medicine—after abnormal fetal lung ultrasound findings were detected during prenatal care at primary care facilities. The prenatal diagnosis was confirmed at our institution by fetal ultrasonography at the initial consultation. Throughout prenatal follow-up, ultrasound examinations were performed at intervals of 2 to 3 weeks, and the patients were monitored until delivery. Our institutional protocol recommends delivery at term, without a mandatory indication for cesarean section in cases of fetal pulmonary malformation, unless maternal or fetal complications arise.

After birth, all neonates with a prenatal diagnosis of CPAM or BPS underwent chest radiography and ultrasonography within the first 24 h of life. Asymptomatic patients were followed on an outpatient basis and underwent contrast-enhanced chest computed tomography (CT) between 3 and 6 months of age for surgical planning; symptomatic patients underwent preoperative contrast-enhanced CT and underwent surgery shortly after birth. An experienced pathologist examined the resected lesions to confirm the diagnosis of CPAM or BPS and classified CPAM according to the Stocker criteria into types 0 to IV, depending on the affected lung site, cyst size, and cyst appearance [11].

Our institution, like several other international centers, recommends surgical resection of CPAM and BPS even in asymptomatic children; this therapeutic option was offered to the parents of all children with lesions confirmed after birth. Following counseling, parents could choose whether to proceed with surgery, provided their children continued outpatient follow-up.

Data were collected in an Excel spreadsheet (Microsoft Corp., Redmond, WA, USA). Quantitative variables were described using means and standard deviations or medians and interquartile ranges, depending on the data distribution. Variable distributions were assessed by visual inspection of histograms with superimposed normal curves and by examining standard deviations relative to the corresponding means. Categorical variables were described using absolute and relative frequencies. Means were compared using Student’s *t*-test. For skewed distributions, the Mann–Whitney *U* test was used. Categorical variables were compared using the chi-square test or Fisher’s exact test. For polytomous variables, adjusted residuals were analyzed when a statistically significant result was obtained to identify specific associations. The significance level was 5% (*p* < 0.05), and the analyses were conducted using SPSS software, version 27.0.

## Results

**Fig. 1:**
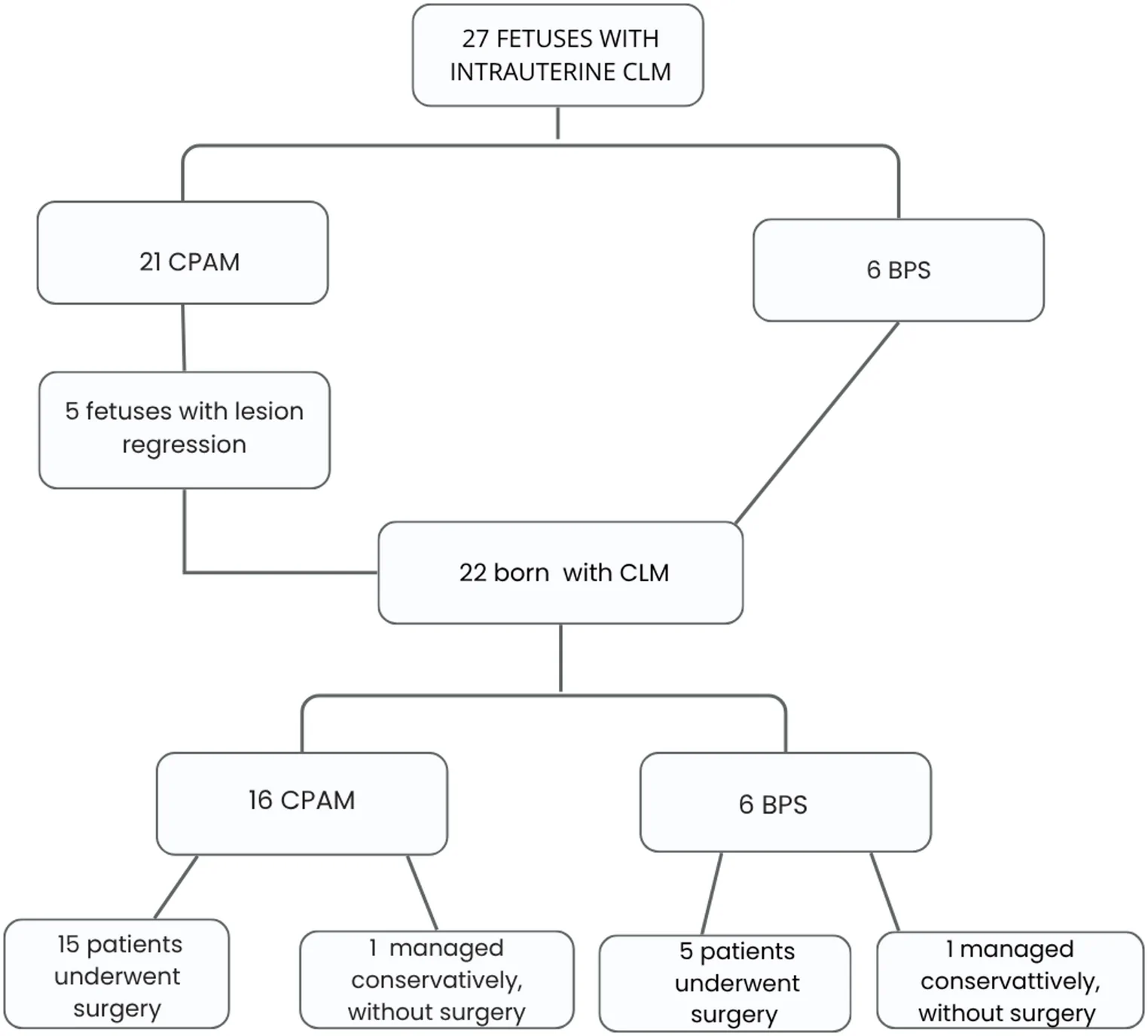
Study flow chart (CLM-congenital lung malformation; CPAM-congenital pulmonary airway malformation; BPS-bronchopulmonary sequestration)

During the study period, the incidence of CPAM was 1 in 1,632 births and the incidence of BPS was 1 in 7,185 births. Among the 27 fetuses prenatally diagnosed with CPAM or BPS, five showed complete regression of their lesions during pregnancy; prenatal ultrasonography suggested CPAM in all five fetuses. In the remaining 22 patients, the prenatal diagnosis of CPAM (16 patients) or BPS (6 patients) was confirmed after birth. The flowchart showing patient inclusion, prenatal lesion regression, postnatal diagnostic confirmation, and surgical treatment is presented in Fig. 1.

The mean gestational age at diagnosis by fetal ultrasonography was 24.3 ± 2.7 weeks. Mediastinal shift was observed on prenatal ultrasonography in eight fetuses (36%). Only two pregnant women underwent abdominal magnetic resonance imaging (MRI) to assess the size and effects of the pulmonary malformation. None of the fetuses required maternal corticosteroid therapy, lesion puncture and aspiration, or the placement of a thoracoamniotic shunt for nonimmune fetal hydrops. Of the 22 patients with a pulmonary lesion diagnosed at birth, 12 (54.5%) were delivered vaginally and 10 (45.5%) by cesarean section; the median Apgar score was 8 (7–9) at one minute and 9 (9–9) at five minutes, and the mean birth weight was 2,968 (± 582) g. The mean maternal age was 27.9 (± 7) years, and the mean gestational age at birth was 37.5 (± 2.6) weeks.

The clinical profile and surgical data of the 22 patients with persistent lesions are presented in Table 1. In this group, prenatal ultrasonographic findings suggestive of CPAM in 16 patients and BPS in six were confirmed by chest CT. Respiratory symptoms were detected shortly after birth in 10 out of 22 patients (45.5%); 12 (54.5%) were asymptomatic. Among the symptomatic patients, seven (70%) had tachypnea and two (20%) developed respiratory distress syndrome. One (10%) newborn with ventilatory failure requiring mechanical ventilation also had congenital chylothorax on the same side of the pulmonary malformation [12].

Malformations affected the left lung in 12 newborns (54.5%) and the right lung in 10 (45.5%). Among the 16 patients with CPAM, CVR was determined in five, with a median value of 1.32 (0.39 to 1.52). Associated malformations were observed in two (9%) children diagnosed with CPAM: one with congenital chylothorax and the other with congenital heart disease. The parents of two asymptomatic newborns declined surgery, and thus 20 patients underwent surgical treatment.

Among the 20 patients who underwent surgery, 10 (50%) were male. The median age at surgery was 5.5 (4 to 8) months, with a mean weight of 7.57 (± 2.7) kg. Pulmonary lobectomy was performed by video-assisted thoracoscopy in 15 cases (75%) and by conventional thoracotomy in five cases (25%). The anatomopathological diagnosis was CPAM in 15 (75%) patients and BPS in five (25%). CPAM was classified as type I in 10 patients, type II in four, and type IV in one (Table 1). No hybrid lesions or type III CPAM were observed.

Postoperative complications occurred in five out of 20 operated newborns (25%), including two cases of surgical wound infection (10%), one after video-assisted thoracoscopy and the other after thoracotomy. We also observed one case of pneumothorax and one case of esophageal perforation, both of which were related to video-assisted thoracoscopy surgeries, which were managed with replacement of the chest tube and the use of an intraesophageal vacuum dressing respectively [13]. There were no deaths in the operated group. The operated newborns evolved well and remain asymptomatic and without complications at a median follow-up of 18 months (10 to 33.5). The two patients who presented malformations at birth and whose parents declined surgery were followed up and remain asymptomatic after 9 and 24 months.

Statistical analyses comparing patients according to symptom status (symptomatic versus asymptomatic), diagnosis (CPAM versus BPS), and surgical approach (VATS versus open surgery) revealed no significant differences between the groups.

**Table 1:** Characteristics of newborns with pulmonary malformation at birth

ID	Sex	Respiratory symptoms	Affected lobe	Age at surgery (months)	Lobectomy approach	Lesion type	Surgical complication
1	F	No	RLL	4	VATS	CPAM - II	No
2	M	Yes	LLL	-	No	CPAM - I	–
3	F	Yes	RLL	5	VATS	CPAM - I	No
4	M	No	LLL	24	VATS	BPS	No
5	F	Yes	LLL	-	No	BPS	–
6	M	No	LLL	8	VATS	CPAM - II	No
7	M	Yes	RUL	6	Open	CPAM- IV	No
8	F	Yes	RUL	5	VATS	CPAM - I	Pneumothorax
9	M	Yes	RLL	0,3	Open	CPAM - II	No
10	M	No	RLL	9	Open	BPS	No
11	F	Yes	LLL	0,5	Open	BPS	SW Infection
12	F	No	LUL	5	VATS	CPAM - I	No
13	M	No	RLL	5	VATS	CPAM - I	No
14	M	No	RLL	4	VATS	CPAM - I	No
15	F	No	ML	4	VATS	CPAM - II	No
16	M	No	RLL	6	VATS	CPAM - I	Esophageal perforation
17	M	No	LLL	8	VATS	CPAM - I	No
18	F	No	LLL	19	VATS	CPAM - I	No
19	M	Yes	LLL	1	Open	BPS	No
20	F	No	LUL	6	VATS	CPAM - I	No
21	F	Yes	LLL	8	VATS	CPAM - I	SW Infection
22	F	Yes	LLL	15	VATS	BPS	No

Note: *M* male, *F* female, *RLL* right lower lobe, *LLL* lower left lobe, *RUL* right upper lobe, *LUL* left upper lobe, *ML* medium lobe, *CPAM* congenital pulmonary airway malformation, *BPS* bronchopulmonary sequestration, *VATS* videothoracoscopy, *SW* surgical wound

## Discussion

In this retrospective study conducted at a quaternary referral hospital, the incidence of CPAM and BPS was much higher than that reported in the literature. This may reflect our hospital’s status as a center of excellence in fetal medicine, to which pregnant women from several states are referred for follow-up when a fetal malformation is detected by ultrasonography during prenatal care [3, 14, 15]. As previously reported [16], CPAM was the most common lesion in our sample, consistent with our finding that all lesions that regressed during pregnancy had the ultrasonographic appearance of CPAM.

Of the 27 fetuses with CLMs followed during pregnancy, five (18.5%) had complete regression of the lesion before birth, as previously described [17]. It should be noted, however, that lesions observed on prenatal ultrasound that are not visible on chest radiographs after birth must be assessed by chest CT to confirm regression, because many lesions not visualized on chest radiography remain visible on chest CT. In this study, chest CT performed before surgery was an important step in both confirming the prenatal diagnosis and providing anatomical details of the pulmonary malformation.

CLMs are readily detected on routine prenatal ultrasonography performed between 18 and 22 weeks of gestation [18]. They may appear as cystic or solid lesions that occupy space in the fetal thorax or may be suggested by abnormal lung size accompanied by mediastinal deviation [8]. These lesions generally increase in size between 20 and 26 weeks of gestation before reaching a plateau at 29 weeks, when they stop growing or may even decrease in size [19, 20]. On prenatal ultrasonography, CPAM is generally recognized as a multilocular lesion containing cysts or as a hyperechoic solid mass, with deviation of the heart to the contralateral side [21], whereas BPS appears as a well-defined hyperechoic mass, often resembling solid CPAM. However, Doppler ultrasonography may reveal a feeding artery originating from the aorta, a finding characteristic of BPS.

A large CPAM may grow and cause mediastinal deviation, with consequent esophageal compression, pulmonary hypoplasia, polyhydramnios, and obstruction of venous return, which may lead to hydrops fetalis. Therefore, prenatal monitoring of CPAM growth through calculation of the CPAM volume ratio (CVR) is recommended. We were able to obtain CVR measurements for only five of our patients with CPAM, and the values were below 1.6 in all cases. A CVR below 1.6 is associated with a less than 3% chance of progression to hydrops fetalis and a fetal survival rate of 94% [9, 22]. The low CVR values in our sample likely explain why none of our patients with prenatal CPAM developed hydrops. Accordingly, none of the patients with macrocystic lesions required cyst decompression by thoracentesis or insertion of a thoracoamniotic shunt, and none of those with microcystic lesions required maternal treatment with betamethasone. Only two patients (9%) had other malformations associated with CPAM, a proportion consistent with the 11% reported in the literature [9, 22].

The literature indicates that most children born with CPAM or BPS are asymptomatic during the neonatal period [23]. In our sample, approximately half of the patients had respiratory symptoms at birth, most commonly tachypnea. One newborn with CPAM had respiratory symptoms associated with an ipsilateral pleural effusion (congenital chylothorax) and required surgery during the neonatal period to resect the lesion and treat the chylothorax [12]. Conversely, about half of the patients who underwent surgery were asymptomatic at the time of the procedure. Although surgery is recommended for symptomatic children, its role remains controversial in asymptomatic patients [24]. Nevertheless, reports indicate that approximately 80% of individuals between 15 and 80 years of age with these malformations experience cough and recurrent pulmonary infections [25]. Furthermore, when these lesions are diagnosed in adults, they are routinely resected, regardless of the presence of symptoms [9]. Therefore, we favor prophylactic surgical resection to prevent future infections, continued lesion growth, and potential malignant transformation [9, 22, 26].

To date, there have been no well-designed long-term follow-up studies of neonates with asymptomatic CPAM or BPS into adulthood; monitoring our two asymptomatic patients who did not undergo surgery is therefore important to ensure their well-being. In individuals with CPAM or BPS, infections may complicate surgical resection, increasing procedural difficulty and the risk of complications and conversion to conventional open thoracotomy. The risk of malignant transformation in CPAM and BPS is unknown, although it has been estimated to be 2–4% [27, 28]. The tumors most commonly associated with these malformations are pleuropulmonary blastoma and adenocarcinoma [29].

When surgical resection of CPAM or BPS is indicated, video-assisted thoracoscopic surgery is the preferred approach. Video-assisted pulmonary resection has demonstrated advantages over conventional thoracotomy [30]. Video-assisted thoracoscopy is a minimally invasive procedure that provides better visualization of anatomical structures and is feasible in children of any age or weight. Because it is less invasive, video-assisted thoracoscopic lobectomy results in less postoperative pain, shorter hospital stays, and lower long-term morbidity, including a lower risk of chest wall deformities and scoliosis resulting from the muscle incisions required for thoracotomy [31].

The timing of surgical resection of asymptomatic CPAM or BPS is also a matter of debate. Although resection is elective in these cases, some surgeons prefer to operate early, at around 4–6 months of age, whereas others prefer to wait until the child is 1 year old. Procedures performed at a later age have not demonstrated better surgical outcomes [30]. In addition, older children may develop inflammation and lymphadenopathy in and around the pulmonary arteries, which can complicate the identification and ligation of blood vessels. Data suggest that early surgery is associated with shorter operative times and hospital stays and a lower incidence of inflammation in the resected pulmonary lobe. Early resection also reduces the likelihood of preoperative respiratory infection, which can alter surgical anatomy, create adhesions, and complicate the procedure, thereby increasing the likelihood of conversion from a thoracoscopic approach to conventional open thoracotomy. Evidence from the literature indicates that patients undergoing early resection have favorable outcomes, with no deaths and only minor postoperative complications that can be managed effectively [30].

This study has some limitations, including its retrospective design, small sample size, short follow-up period, and the availability of prenatal CVR measurements in only five cases, which may have influenced the assessment of fetal risk. However, large samples are difficult to obtain because these congenital lesions are rare and often remain undetected, and comparative multicenter studies may be difficult to design. Children undergoing surgery for a congenital lung malformation should ideally be followed until at least 8–10 years of age to assess lung growth and compensatory development and to monitor for possible late complications. Because our study spanned only 11 years, we were unable to obtain follow-up data for all children who underwent surgery. Prenatal CVR measurements were available in only five cases because CVR assessment was not part of the clinical protocol when the first patients were evaluated. Despite these limitations, our data underscore the importance of prenatal diagnosis of CPAM and BPS, which allows pregnant women to be referred to a specialized center for appropriate care.

## Conclusions

Prenatal diagnosis of CPAM and BPS is essential for family counseling and referral of pregnant women to specialized facilities for the management of these malformations. Although some lesions may regress before birth, most persist. Clinical monitoring is an option for asymptomatic patients; however, we prefer surgical resection to reduce the risk of future pulmonary infections and potential malign transformation. When indicated, surgery should preferably be using a video-assisted thoracoscopic approach.

## Data Availability

No datasets were generated or analysed during the current study.
